# Prevalence and factors associated with fertility desire among people living with HIV: A systematic review and meta-analysis

**DOI:** 10.1371/journal.pone.0248872

**Published:** 2021-03-18

**Authors:** Xiang Yan, Jie Du, GuoPing Ji

**Affiliations:** 1 Department of Epidemiology and Health Statistics, School of Public Health, Anhui Medical University, Hefei, Anhui, China; 2 The First Affiliated Hospital of USTC, Hefei, Anhui, China; 3 Anhui Provincial Center for Women’s and Children’s Health, Hefei, Anhui, China; Makerere University School of Public Health, UGANDA

## Abstract

**Background:**

The fertility desire of people living with HIV (PLHIV) has been rising in the past decade. However, there are many studies among which the association remains controversial between the fertility desire of HIV-infected persons and antiretroviral therapy (ART), sex, marital status, and educational level.

**Methods:**

We performed a literature search of these meta-analyses in PubMed, the Cochrane Library, Web of Science and ScienceDirect in November 2019. We also reviewed references of eligible studies to complement the search. We used pooled odds ratios (ORs) and 95% confidence intervals (CIs) with a random-effects model and a fixed-effects model to estimate the association between fertility desire among PLHIV and ART, sex, age, marital status, educational level, and number of children. Subgroups with I square values (I^**2**^) and sensitivity analyses were performed to assess the heterogeneity and the stability of the overall ORs, respectively. We evaluated publication bias using Egger’s test and a visual inspection of the symmetry in funnel plots.

**Results:**

In these meta-analyses 50 articles were included with 22,367 subjects. The pooled prevalence of fertility desire among PLHIV was estimated to be 42.04%. The pooled analyses showed that the fertility desire of PLHIV is associated with ART (OR = 1.11, 95% CI:1.00–1.23, P = 0.043), sex (OR = 1.51, 95% CI:1.10–2.09), age (OR = 2.65, 95% CI:2.24–3.14), marital status (OR = 1.34, 95% CI:1.08–1.66), educational level (OR = 0.85, 95% CI:0.73–1.00, P = 0.047) and the number of children (OR = 3.99, 95% CI:3.06–5.20). PLHIV who are on ART, are male, are younger than 30, are married/cohabiting, have received a secondary education or above, and are childless have a higher prevalence of fertility desire. The two factors of age and the number of children, in particular demonstrated a strong significant association with fertility desire. We found moderate heterogeneity in the meta-analyses of age and educational level and high heterogeneity in the meta-analyses of sex, marital status and number of children. Publication bias was detected in the meta-analyses of the association of fertility with sex and educational level.

**Conclusion:**

This study demonstrates that the prevalence of fertility desire among HIV-infected people is 42.04%, and the fertility desire among PLHIV is associated with ART experience, sex, age, marital status, the number of children, and educational level. Since a majority of PLHIV are of reproductive age, it is necessary to support PLHIV in terms of their needs regarding reproductive decision-making. Through counseling and reproductive health care, further measures to prevent the horizontal and vertical transmission of HIV should be taken.

## Introduction

According to the 2019 reports of Joint United Nations Programme on HIV/AIDS (UNAIDS), there were approximately 37.9 million people living with HIV (PLHIV) across the world, among whom 1.47 million were of reproductive age [1]. It is common for them to want to get married and start a family. However, the prevalence of fertility desire/intention among PLHIV has been low for the past two decades due to poor health status, fear of infecting one’s spouse or fetus, and discouraging policies in many countries [2]. In 2000, a study conducted in Europe showed that the pregnancy rate trended to decrease in women after receiving a diagnosis of HIV while the rate of abortion had increased [3]. Lewis et al showed that HIV-infected women in Sub-Saharan Africa have a lower fertility rate than their non-infected counterparts [4].

With more access to highly active antiretroviral therapy (HAART), nevertheless, the quality of life for PLHIV has significantly improved, and their life span is expected to be longer [5, 6]. As a result, their fertility desire or intention has risen [7, 8]. A study from Ethiopia indicated that the prevalence of fertility desire among HIV-positive individuals increased from 20.0% in 2010 to 42.1% in 2013 [9, 10]. A similar growing tendency toward the desire to reproduce has been found in other regions as well [11]. A number of studies have pointed to many factors that could influence the fertility desire of PLHIV. For example, HIV-positive individuals who are young tend to have a higher prevalence of fertility desire [12, 13]. Decreased fertility desire is related to divorce or separation compared to being married, and to having at least one child in contrast to having no children [12–14]. Several studies have revealed that educational status is a predicator of fertility desire [15]. In addition, HIV disclosure to sexual partners could affect the fertility desire of HIV-infected people [14, 16].

Since the fertility desires of PLHIV is tied to sexual practices and pregnancy, it is crucial to prevent horizontal transmission between partners and mother-to-child transmission (MTCT). Previous studies indicate that the viral load of PLHIV on ART could be suppressed; therefore, the risk of transmitting HIV to sexual partners could decrease or even be eliminated [17]. Many measures for sero-discordant couples, such as artificial insemination, timed unprotected intercourse, assisted reproductive techniques, sperm washing and pre-exposure prophylaxis, could be used to reduce HIV horizontal transmission [18–20]. There are also many means of preventing the mother-to-child transmission of HIV (PMTCT), including HIV testing and counseling, the use of ARV drugs, safe delivery, and safe breastfeeding. As a consequence, fertility desire among PLHIV not only has significant implications for such individuals, their partners, and fetuses, but also plays a critical role in preventing HIV transmission and providing reproductive health care.

Studies have shown that the fertility desire of PLHIV is associated with many sociodemographic factors, while there are many inconsistent conclusions regarding whether being on ART is related to higher fertility desire [21–23]. A previous meta-analysis conducted in 2013 explore the associations of fertility with some variables including ART experience, sex, age, the number of children, and educational level [24]. In that study, however, the results showed no significant association of fertility desire with ART experience and sex and hence might not estimate the true effect size; it should be noted that the association between fertility desire and the factors stated above remains inconsistent. Since this study involves more literature on research performed from 2013 to 2019, the objective is to offer a broad description of fertility desire among PLHIV, and was to underscore the strength of the association between these factors and fertility desire.

## Methods

We reported this meta-analysis with reference to the Preferred Reporting Items for Systematic Review and Meta-Analyses (PRISMA) guidelines [25] (S1 Table).

### Search strategy

The literature searching was conducted in PubMed, Cochrane Library, Web of Science and ScienceDirect by following the search strategy and completed before November 24, 2019 (S2 Table): (Fertility desire OR Fertility intention OR desire to have children OR Reproductive intention OR Reproductive decision making OR Desire for child OR Childbearing desire OR Childbearing intention OR parenthood OR fatherhood OR motherhood OR maternity OR paternity) AND (HIV OR people living with HIV OR HIV-positive OR HIV-infected). We extracted all relevant studies by reading theirs titles, abstracts, keywords and full texts. The references of the included studies and any relevant meta-analyses were also reviewed.

### Inclusion and exclusion criteria

The inclusion criteria were: 1)the outcome of interest was fertility desire or intention in the future; 2) the female subjects in the study were all of childbearing age and able to achieve pregnancy; 3)the studies reported the prevalence of fertility desire among PLHIV or least one of the selected associated factors (sex, age, marital status, educational level, number of children, and ART experience); 4)the details of the sample/subsample could be extracted; 5)observational studies published from 2000 to November 2019; and 6)quantitative studies. Studies were excluded if 1) the study was a review, a qualitive study or only an abstract; 2) the study’s objective was irrelevant; 3) the study’s subjects were ineligible. and 4) data were not available or adequate.

### Data extraction

The following data from the eligible studies were collected: the first author’s name, publication year, the country/location where the study was conducted, the study design, the sample size, the number of men and women, the prevalence of fertility desire among PLHIV, and the study’s quality assessment score. If the adjusted odds ratio (OR) values and their 95% confidence intervals (CIs) were unavailable, we directly extracted or calculated the ORs with corresponding 95% CIs using the raw data. Variables of age, marital status, the number of children and educational level were respectively dichotomized as aged below 30 years vs aged 30 years and above, currently married/cohabiting vs not currently married (single, widowed, separated or divorced), having children vs having one or more children, and up to primary education vs secondary education or above. In this review, two investigators (XY and JD) screened all studies carefully to ensure that they met the inclusion criteria. Inconsistencies were resolved by the chief investigator GPJ if they existed.

### Quality assessment

The quality of the included studies was assessed by using an 11-item checklist for cross-sectional study quality, as recommended by the Agency for Healthcare Research and Quality (AHRQ) [26]. For the 11-item checklist, an item would be scored ‘0’ if an answer of “NO” or “UNCLEAR” was given; if an answer of “YES” was given, then the item scored ‘1’. The definition of article quality was as follows: low quality = 0–3; moderate quality = 4–7; and high quality = 8–11 (S3 and S4 Tables).

### Statistical analysis

First, pooled ORs with 95% CIs were calculated by using the extracted raw data, crude or adjusted ORs with 95% CIs that evaluate the strength of the association between fertility desire/intention among PLHIV and the factors of interest (sex, age, marriage status, educational level, number of children and ART experience). Then six meta-analyses were performed. To assess the robustness of the outcomes, we conducted subgroup analysis in terms of publication year, region, and quality assessment score. Heterogeneity between the studies was assessed by using the Cochran’s Q Chi-square test and I^**2**^ analysis (I^**2**^ values of 50% and 75% were considered moderate and high heterogeneity respectively). When I^**2**^≥50%, we selected a random-effects model for heterogeneity analyses, and an otherwise fixed-effects model. Sensitivity analysis was performed to explore the impact of individual studies on the results. We evaluated publication bias through visual inspection of asymmetry in funnel plots or with the P value of Egger’s test. We carried out all analyses were done with Stata (Version12.0).

## Results

### Study selection

**Fig 1:**
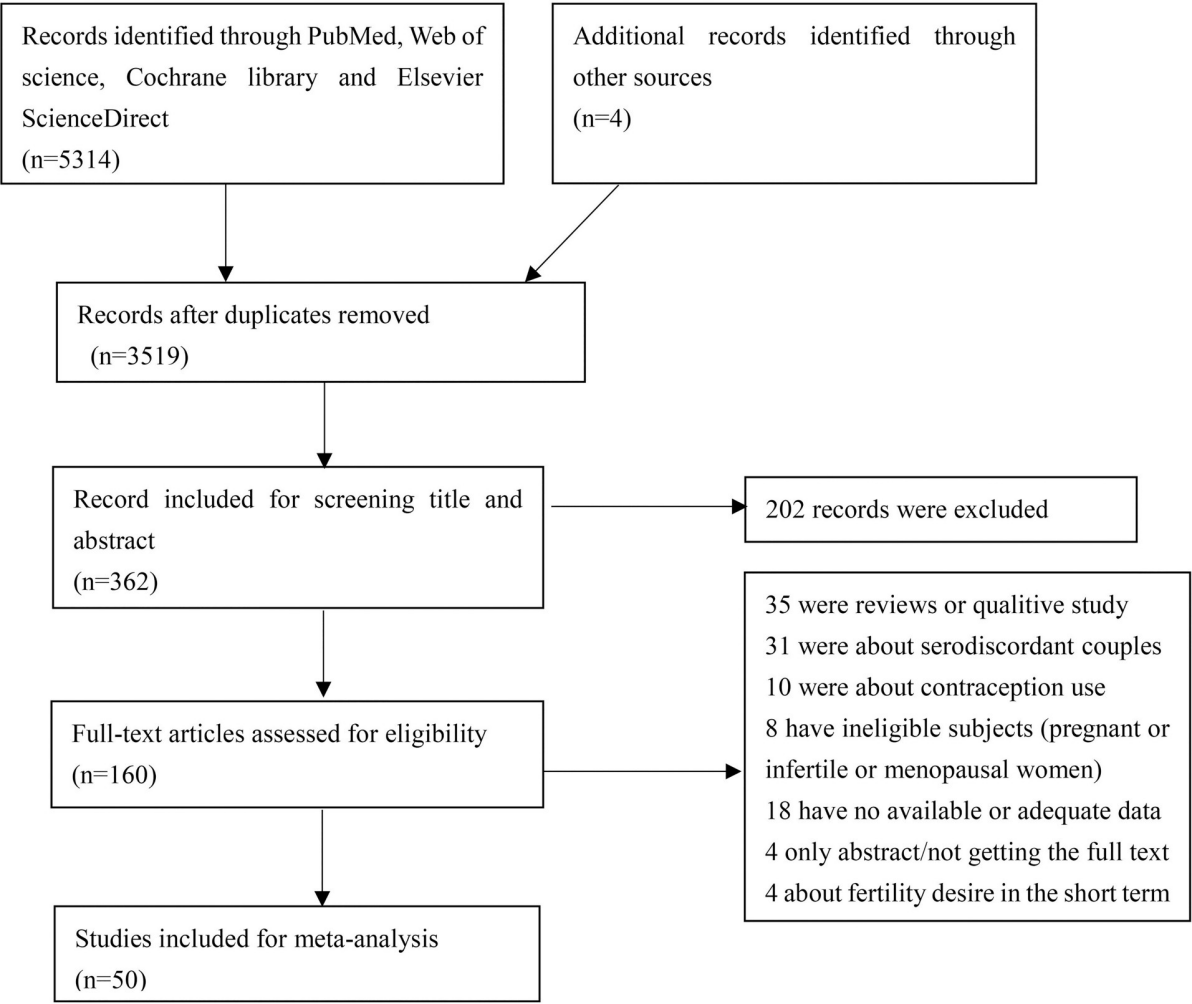
Flowchart of the literature search.

According to the search strategy we retrieved 5,314 articles, of which 1,795 were duplicates. Finally, 50 articles were assessed for eligibility in these meta-analyses after full-text screening [10, 14, 27–74]. A flowchart of the literature search was presented in Fig 1.

### Description of the studies

In these meta-analyses, we included a total of 50 articles with 22,367 participants. All included studies were cross-sectional and published between 2000 and -2019, and scored no less than 6 points on the basis of quality assessment. According to the income group defined by the World Bank for 2017, the study countries were categorized as low income, lower middle income, upper middle income, and high income [75]. Among these studies, 20 were conducted in low-income countries (LICs), 12 in lower middle-income countries (LMICs), 11 in upper middle-income countries (UMICs) and 7 in high-income countries (HICs). The general information is shown in Table 1.

**Table 1:** The main features of the studies included in the meta-analysis.

First author	Publication year	Location	Country’s income level	Study design	Sample size	Number of men/women	Fertility desire (%)	Study quality score
Abbawa, F.	2015	Ethiopia	Low	cross-sectional	422	217/205	141(33.41%)	7
Adilo, T. M.	2017	Ethiopia	Low	cross-sectional	416	124/292	227(54.57%)	7
Adler, D. H.	2017	South Africa	Upper middle	cross-sectional	50	0/50	40(80.00%)	7
Alemayehu B	2012	Ethiopia	Low	cross-sectional	307	185/122	203(66.12%)	7
Asfaw, H. M.	2014	Ethiopia	Low	cross-sectional	1855	0/1855	815(43.94%)	6
Cohn, S. E.	2018	USA	High	cross-sectional	1425	1181/244	580(40.70%)	8
Cooper, D.	2009	South Africa	Upper middle	cross-sectional	459	174/285	148(32.24%)	9
de Souza, M. R.	2017	Brazil	Upper middle	cross-sectional	274	0/274	71(25.91%)	7
Demissie, D. B.	2014	Ethiopia	Low	cross-sectional	340	126/214	133(39.12%)	7
Finocchario Kessler, S.1	2010	USA	High	cross-sectional	181	0/181	107(59.12%)	8
Finocchario Kessler, S.2	2014	Brazil	Upper middle	cross-sectional	295	295/0	115(38.98%)	8
Erhabor, O.	2012	Nigeria	Lower middle	cross-sectional	195	88/107	111(56.92%)	6
Gyimah, A. A.	2015	Ghana	Lower middle	cross-sectional	295	0/295	172(58.31%)	8
Haddad, L. B.	2016	USA	High	cross-sectional	181	0/181	62(34.25%)	7
Heard, I.	2007	France	High	cross-sectional	1254	699/555	322(25.68%)	9
Hernando, V.	2014	Spain	High	cross-sectional	134	0/134	66(49.25%)	9
Iliyasu, Z.	2009	Nigeria	Lower middle	cross-sectional	340	85/255	219(64.41%)	8
Jose, H.	2016	India	Lower middle	cross-sectional	230	132/98	77(33.48%)	8
Kaida, A.	2011	South Africa	Upper middle	cross-sectional	432	0/432	130(30.09%)	9
Kawale, P.	2014	Malawi	Low	cross-sectional	202	75/127	103(50.99%)	9
Kipp, W.	2011	Uganda	Low	cross-sectional	199	77/122	27(13.57%)	9
Krashin, J. W.	2018	Malawi	Low	cross-sectional	558	250/308	175(31.36%)	8
Laar, A. K.	2015	Ghana	Lower middle	cross-sectional	318	0/318	135(42.45%)	7
Laryea, D. O.	2014	Ghana	Lower middle	cross-sectional	230	0/230	123(53.48%)	7
Litwin, L. E.	2015	Uganda	Low	cross-sectional	436	0/436	162(37.16%)	9
Maier, M.	2009	Uganda	Low	cross-sectional	501	0/501	73(14.57%)	9
Mayhew, S. H.	2017	Kenya	Lower middle	cross-sectional	234	0/234	66(28.21%)	8
Mekonnen, B.	2019	Ethiopia	Low	cross-sectional	427	0/427	172(40.28%)	9
Mekonnen, H.	2017	Ethiopia	Low	cross-sectional	360	0/360	174(48.33%)	8
Melaku, Y. A.	2014	Ethiopia	Low	cross-sectional	964	0/964	439(45.54%)	9
Mmbaga, E. J.	2013	Tanzania	Lower middle	cross-sectional	410	146/264	152(37.07%)	7
Moyo, W.	2004	Zimbabwe	Lower middle	cross-sectional	2250	0/2250	1210(53.78%)	8
Myer, L.	2007	South Africa	Upper middle	cross-sectional	311	84/227	89(28.62%)	8
Nakayiwa, S.	2006	Uganda	Low	cross-sectional	1092	488/604	174(15.93%)	8
Nedjat, S.	2015	Iran	Upper middle	cross-sectional	400	240/160	161(40.25%)	7
Nobrega, A. A.	2007	Brazil	Upper middle	cross-sectional	229	0/229	91(39.74%)	7
Okome-Nkoumou, M.	2015	Gabon	Upper middle	cross-sectional	422	85/337	329(77.96%)	6
Oladapo, O. T.	2005	Nigeria	Lower middle	cross-sectional	147	52/95	93(63.27%)	9
Paiva, V.	2007	Brazil	Upper middle	cross-sectional	739	206/533	202(27.33%)	7
Pokharel, R.	2018	Nepal	Lower middle	cross-sectional	252	131/121	51(20.24%)	7
Pottinger, A. M.	2019	Jamaica	Upper middle	cross-sectional	251	121/130	166(66.14%)	7
Rhodes, C. M.	2016	USA	High	cross-sectional	100	0/100	44(44.00%)	8
Shiferaw, T.	2019	Ethiopia	Low	cross-sectional	374	0/374	175(46.79%)	9
Sufa, A.	2014	Ethiopia	Low	cross-sectional	456	0/421	192(42.11%)	7
Tamene, W.	2007	Ethiopia	Low	cross-sectional	460	216/244	185(40.22%)	6
Tesfaye L	2012	Ethiopia	Low	cross-sectional	389	171/218	164(42.16%)	8
Thomson, K. A.	2018	USA	High	cross-sectional	100	72/28	43(43.00%)	7
Wagner, G.	2012	Uganda	Low	cross-sectional	233	94/139	103(44.21%)	8
Wagner, G. J.	2013	Uganda	Low	cross-sectional	767	261/506	237(30.90%)	8
Wekesa, E.	2014	Kenya	Lower middle	cross-sectional	463	193/270	157(33.91%)	9

### Meta-analysis of fertility desire

**Fig 2:**
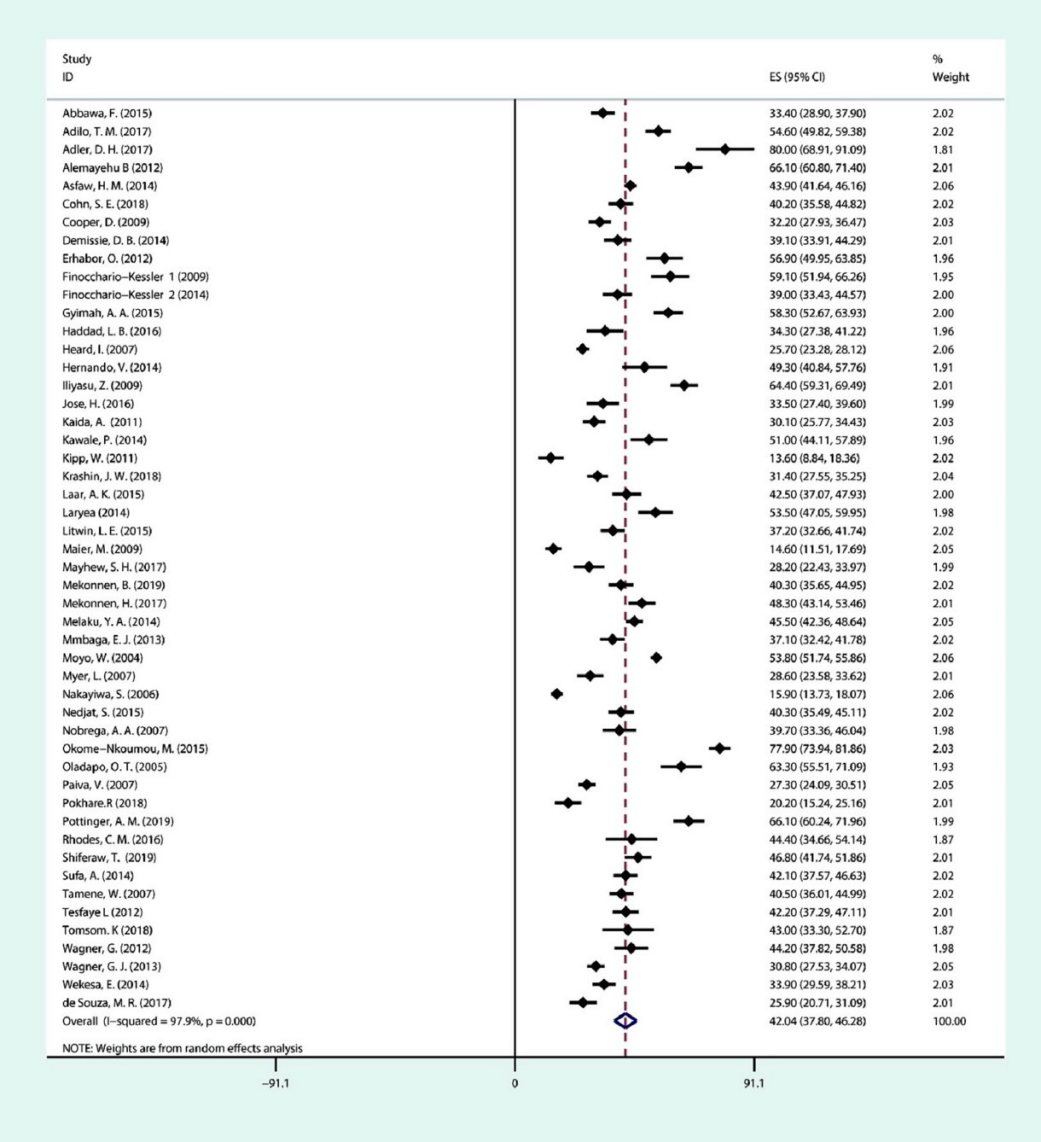
Forest plot of the overall prevalence of fertility desire among people living with HIV.

The prevalence of fertility desire among PLHIV was 13.57% to 80.00% in the fifty included studies (Table 1). The meta-analysis revealed that the prevalence of fertility desire was 42.04%(95% CI: 37.80, 46.28%), with high heterogeneity (*I*^***2***^ = 97.9%, *P* <0.001) using the random-effects model (Fig 2).

**Fig 3:**
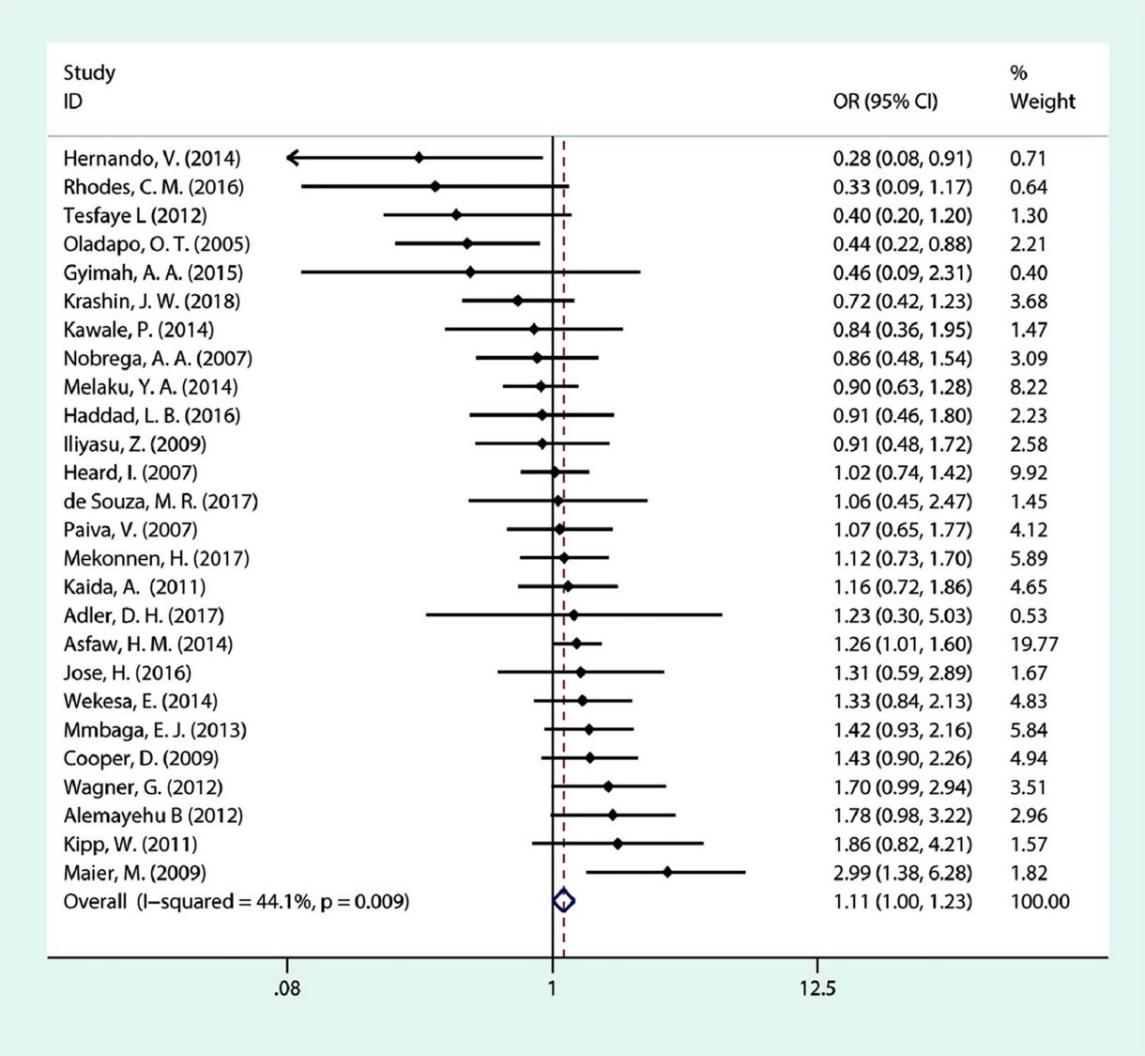
Forest plot of pooled OR for fertility desire in PLHIV (ART experienced vs ART naive).

We have reported the meta-analyses of the associated factors. As portrayed in Fig 3, in this meta-analysis, we have included 26 studies, five of which showed a statistically significant association between ART and fertility desire/intention. As a result, the pooled OR indicated that the fertility desire of PLHIV is statistically and significantly associated with ART (OR = 1.11; 95% CI:1.00–1.23; *P* = 0.043). The testing for heterogeneity did not reveal variability among the included studies (*I*^***2***^ = 44.1%, *P* = 0.009).

**Fig 4:**
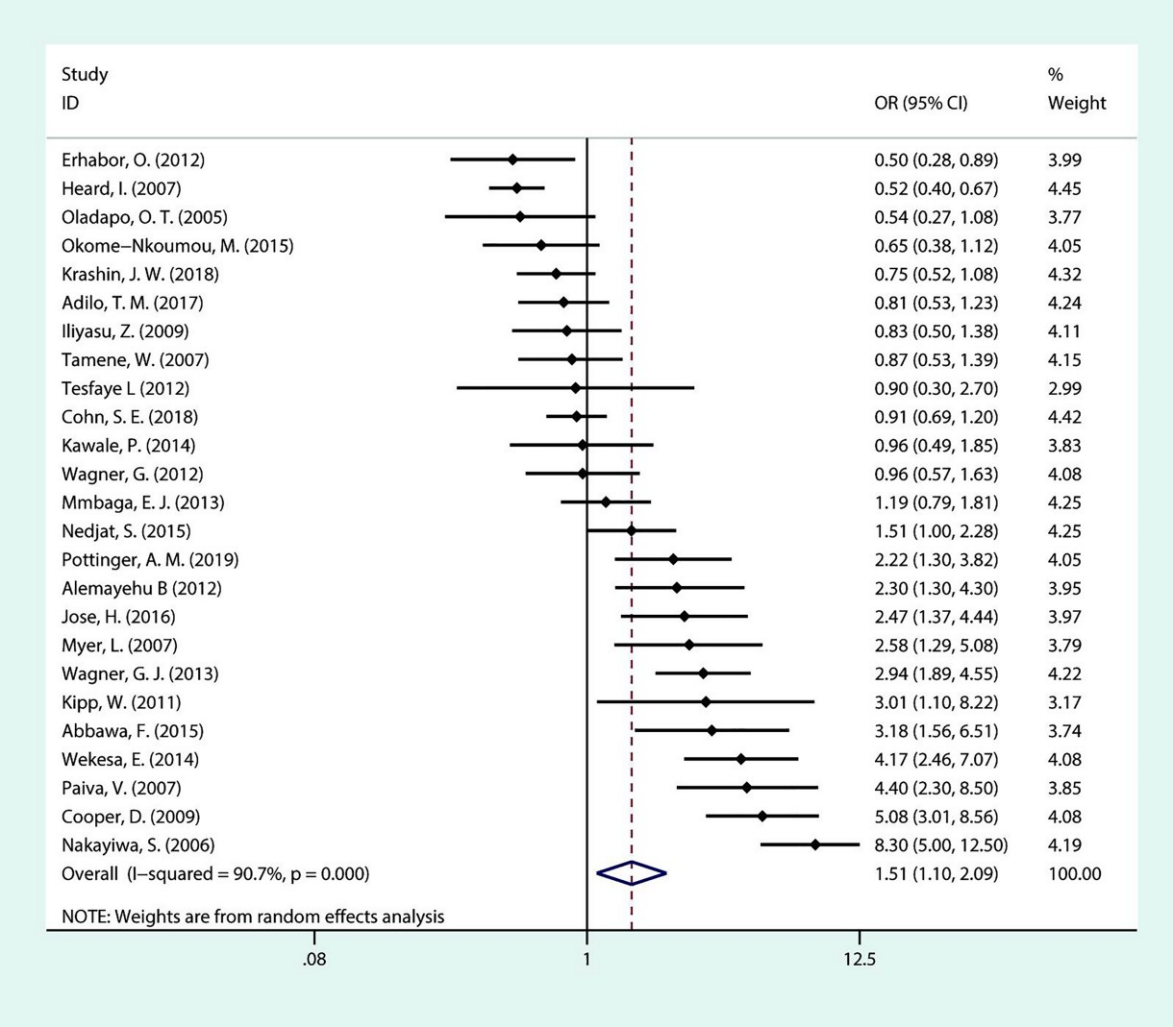
Forest plot of pooled OR for fertility desire in PLHIV (men vs women).

As presented in Fig 4, twenty-five studies were included in this meta-analysis. Thirteen studies showed statistically significant association between sex and fertility desire/intention. Eleven suggested that men have more fertility desire than women while the other two implied the opposite. In terms of sex, the overall OR indicated that men have higher fertility desire than women (OR = 1.51; 95% CI:1.10–2.09). It should be noted that heterogeneity among the included studies was high (*I*^***2***^ = 90.7%, *P* <0.001).

**Fig 5:**
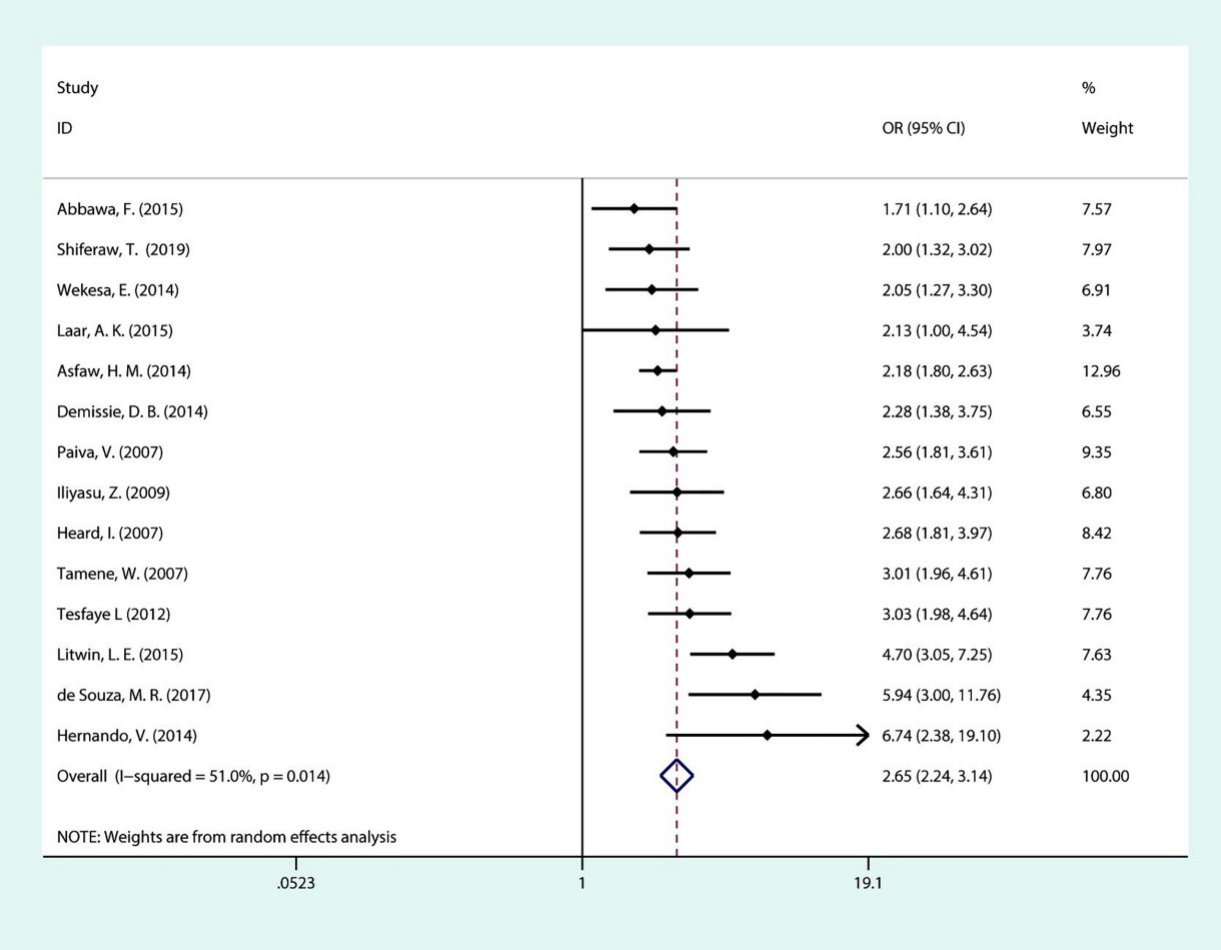
Forest plot of pooled OR for fertility desire in PLHIV (aged below 30 years vs aged 30 and above).

As seen in Fig 5, we included fourteen studies in the meta-analysis of the association between fertility desire and age. It is evident that being younger than 30 is a strong predictor of fertility desire among PLHIV, as almost all ORs with 95% CIs of the included studies fell on the side of increased fertility desire. Similarly, the pooled OR with moderate heterogeneity (*I*^***2***^ = 51.0%, *P* = 0.014), showed that PLHIV younger than 30 years have a 2.6-fold increase in fertility desire compared to their older counterparts (OR = 2.65; 95% CI:2.24–3.14)

**Fig 6:**
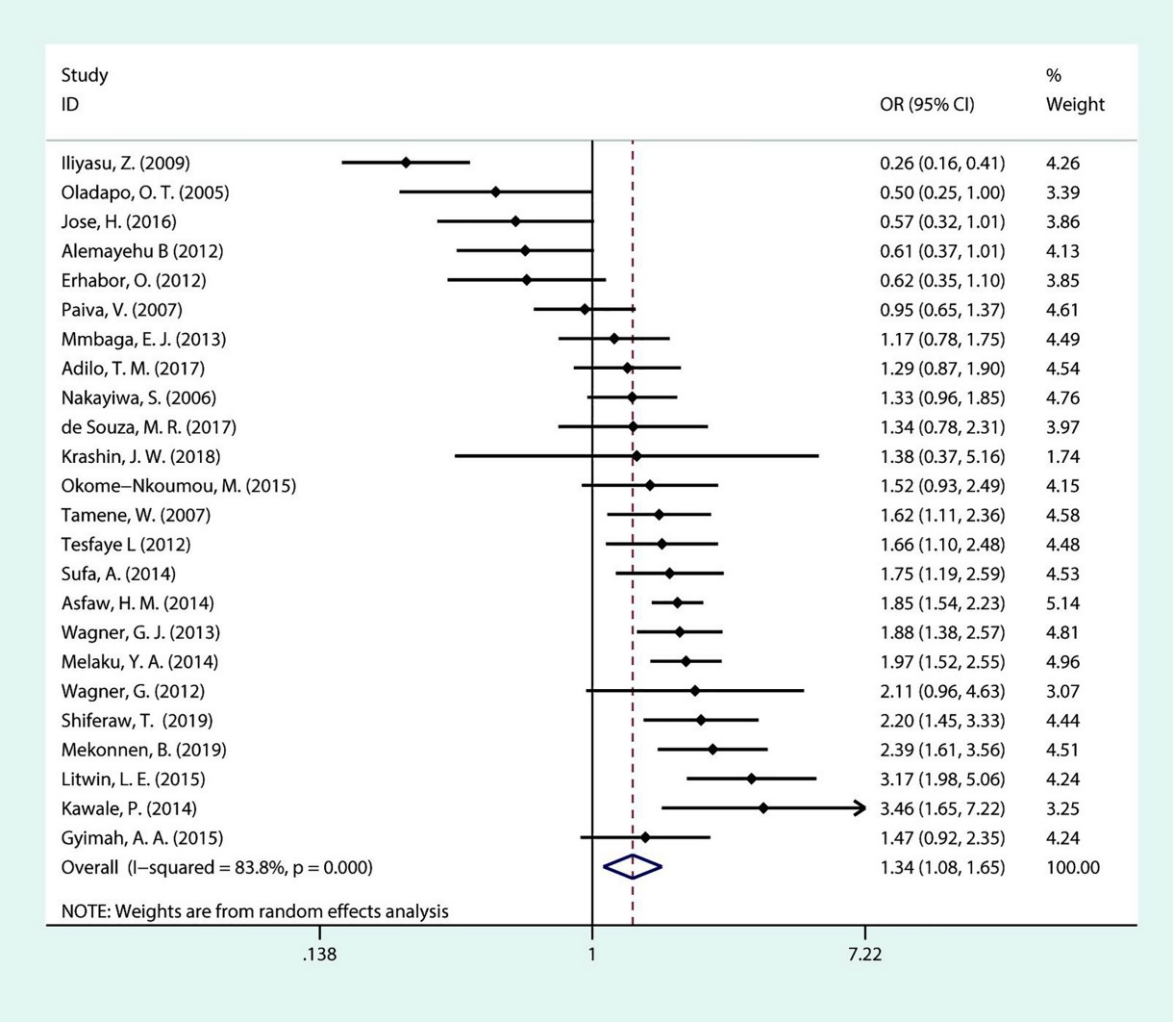
Forest plot of pooled OR for fertility desire in PLHIV (currently married vs unmarried).

As outlined in Fig 6, a meta-analysis including 24 studies was performed to assess association between marriage status and fertility desire. Among the 11 studies that revealed a significant association, only one indicated that PLHIV who are married/cohabiting have less fertility desire than PLHIV who are not married (single, widowed, divorced, or separated). The overall OR demonstrated a positive association of fertility desire with being married/cohabiting (OR = 1.34; 95% CI:1.08–1.65). However, we noted the high heterogeneity (*I*^***2***^ = 83.8%, *P*<0.001).

**Fig 7:**
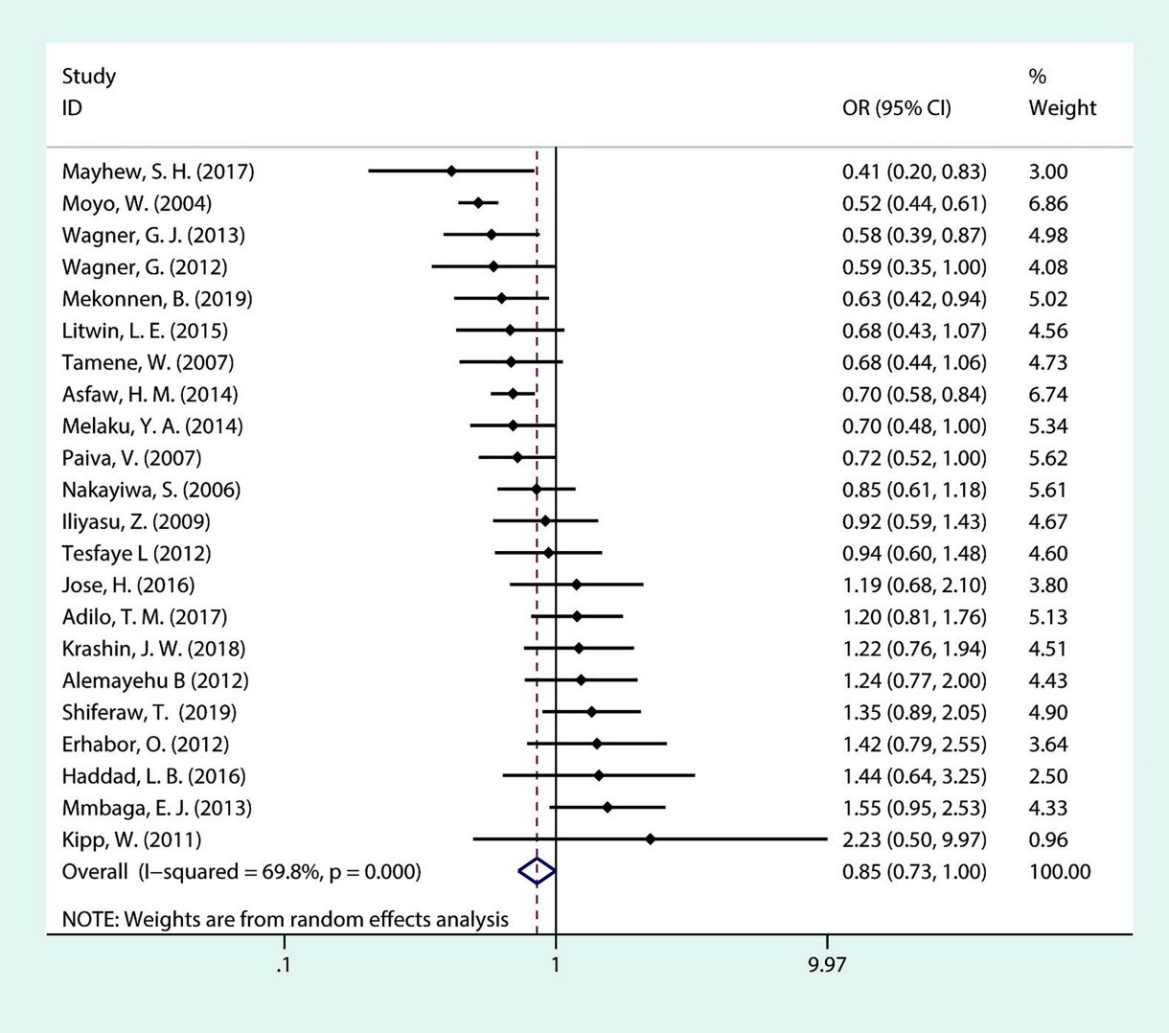
Forest plot of pooled OR for fertility desire in PLHIV (up to primary vs secondary or above).

As portrayed in Fig 7, we included twenty-two studies in the meta-analysis. Five studies consistently found that educational level is associated with fertility desire among PLHIV. The pooled OR implied that, with moderate heterogeneity among the included studies (*I*^***2***^ = 69.8%, *P* = <0.001), PLHIV with a level of up to primary education have less fertility desire than PLHIV whose educational level is secondary or above (OR = 0.85; 95% CI: 0.73–1.00).

**Fig 8:**
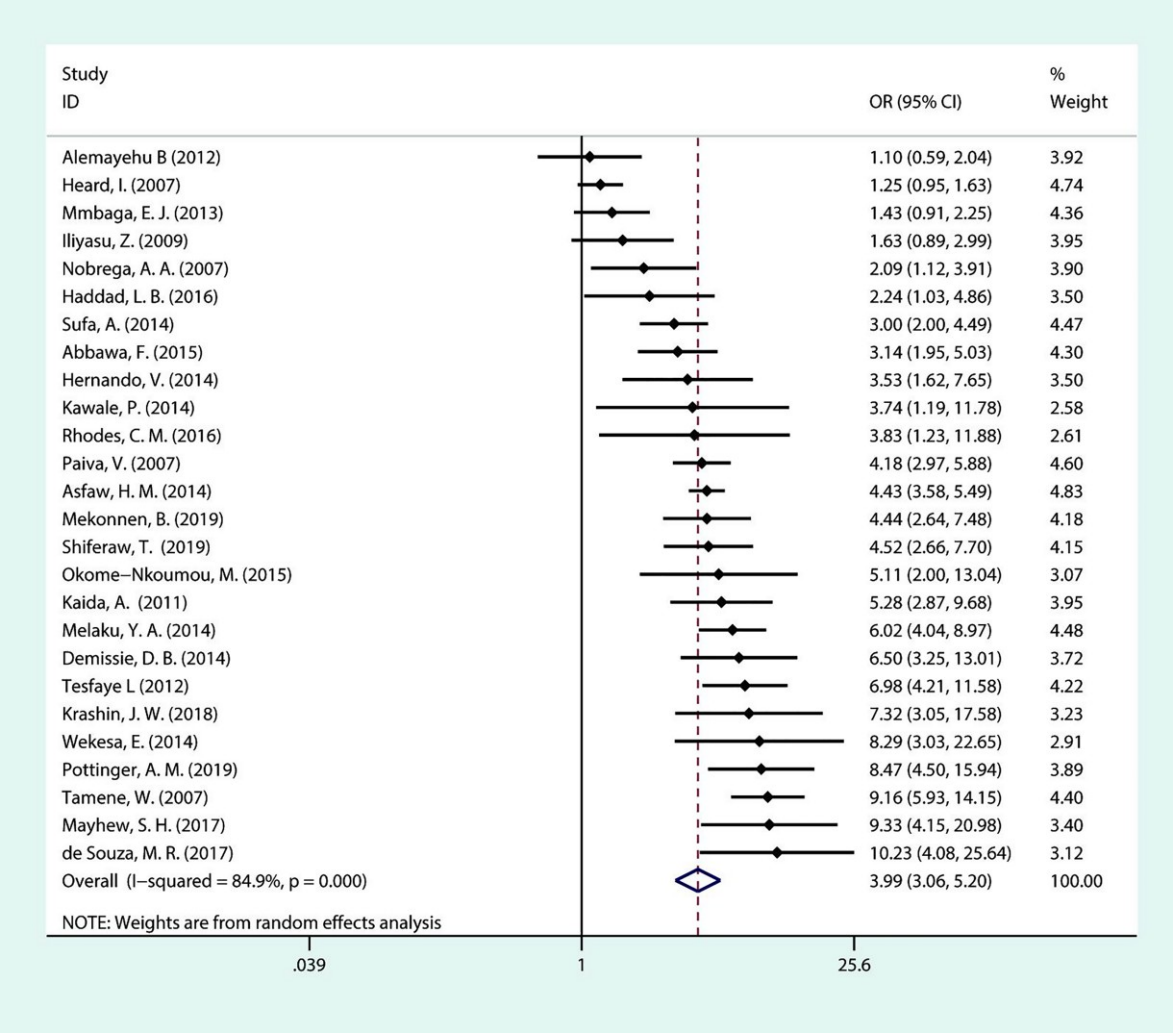
Forest plot of pooled OR for fertility desire in PLHIV (having no children vs having one or more children).

As displayed in Fig 8, there were 26 included studies, of which only four showed no significant association between the number of children and fertility desire in the meta-analysis. As a result, having no children is another strong predictor of fertility desire among PLHIV. The pooled OR indicated that PLHIV who are childless have a nearly 4-fold increase in fertility than PLHIV who have one or more children (OR = 3.99; 95% CI:3.06–5.20). There was high heterogeneity among the included studies (*I*^***2***^ = 84.9%, *P*<0.001).

### Subgroup analysis

Next, we performed subgroup analysis on the basis of publication year, the region where the study was done, and the quality assessment score of the studies. We found associations of fertility desire with age and with the number of children in all subgroups that people living with HIV who were younger than 30 and had no children experienced greater fertility desire. There were relationships between sex and fertility desire shown in studies from Africa (OR = 1.54; 95% CI: 1.04–2.30; *I*^***2***^ = 90.3%). In the subgroup of marriage status, studies published after 2014 (OR = 1.75; 95% CI: 1.44–2.12; *I*^***2***^ = 63.6), those conducted in Africa (OR = 1.42; 95% CI: 1.13–1.77; *I*^***2***^ = 83.9%) and those that scored higher than 7 on the quality assessment (OR = 1.44; 95% CI: 1.02–2.03; *I*^***2***^ = 87.8%) were statistically significant. In light of educational level, we observed an association with fertility desire in studies from Africa as well as in which the studies of which quality assessment score was higher than 7, and the pooled ORs were 0.84 (95% CI: 0.72–0.99, *I*^***2***^ = 72.2%) and 0.78 (95% CI: 0.64–0.95, *I*^***2***^ = 68.3%) respectively. The details of the subgroup analysis were summarized in Table 2.

**Table 2:** The results of the meta-analysis, the heterogeneity test, and publication bias.

Subgroup ^a^	Number of studies	OR (95% CI)	I square (%)	P	P for Egger’s test
**ART**					
Overall	26	1.11(1.00–1.23) ^**b**^	44.1	0.009	0.109
Publication year					
Pre 2014 ^**c**^	13	1.17(0.93–1.48)	57.0	0.006	0.187
Post 2014	13	1.05(0.91–1.22)	23.8	0.203	0.469
Region					
Africa	18	1.14(0.95–1.37)	50.1	0.008	0.457
Other	8	0.95(0.77–1.17)	10.3	0.350	0.089
Quality assessment score					
≤7	8	1.22(1.04–1.44)	0.0	0.717	0.014
>7	18	1.01(0.81–1.25)	55.2	0.003	0.964
**Sex**					
Overall	25	1.51(1.10–2.09)	90.7	<0.001	0.038
Publication year					
Pre 2014	15	1.58(0.96–2.62)	92.9	<0.001	0.183
Post 2014	10	1.40(0.96–2.05)	84.8	<0.001	0.096
Region					
Africa	19	1.54(1.04–2.30)	90.3	<0.001	0.696
Other	6	1.41(0.82–2.42)	90.7	<0.001	0.008
Quality assessment score					
≤7	10	1.37(0.93–2.02)	82.5	<0.001	0.187
>7	15	1.61(1.00–2.59)	93.2	<0.001	0.108
**Age**					
Overall	14	2.65(2.24–3.14)	51.0	0.014	0.082
Publication year					
Pre 2014	5	2.76(2.30–3.32)	0.0	0.967	0.38
Post 2014	9	2.64(2.02–3.47)	67.2	0.002	1.37
Region					
Africa	10	2.41(2.14–2.71)	44.7	0.061	0.527
Other	4	3.47(2.28–5.26)	58.8	0.063	0.054
Quality assessment score					
≤7	7	2.34(2.04–2.68)	47.0	0.079	0.329
>7	7	2.87(2.21–3.73)	52.3	0.049	0.343
**Marital status**					
Overall	24	1.34(1.08–1.65)	83.8	<0.001	0.115
Publication year					
Pre 2014	11	0.99(0.69–1.42)	86.8	<0.001	0.161
Post 2014	13	1.75(1.44–2.12)	63.6	0.001	0.653
Region					
Africa	21	1.42(1.13–1.77)	83.9	<0.001	0.198
Other	3	0.91(0.60–1.39)	56.2	0.102	0.878
Quality assessment score					
≤7	10	1.23(0.97–1.57)	73.7	<0.001	0.02
>7	14	1.44(1.02–2.03)	87.8	<0.001	0.483
**Educational level**					
Overall	22	0.85(0.73–1.00) ^**d**^	69.8	<0.001	0.003
Publication year					
Pre 2014	12	0.84(0.67–1.06)	73.3	<0.001	0.003
Post 2014	10	0.87(0.70–1.09)	64.3	0.003	0.288
Region					
Africa	19	0.84(0.72–0.99)	72.2	<0.001	0.01
Other	3	0.87(0.68–1.14)	48.5	0.143	0.146
Quality assessment score					
≤7	8	0.99(0.78–1.29)	68.2	0.003	0.019
>7	14	0.78(0.64–0.95)	68.3	<0.001	0.027
**Number of child**					
Overall	26	3.99(3.06–5.20)	84.9	<0.001	0.272
Publication year					
Pre 2014	9	2.77(1.60–4.81)	92.4	<0.001	0.53
Post 2014	17	4.64(4.09–5.27)	37.4	0.061	0.299
Region					
Africa	17	4.54(3.53–5.84)	73.0	<0.001	0.878
Other	9	3.11(1.87–5.19)	87.6	<0.001	0.135
Quality assessment score					
≤7	13	3.72(2.66–5.19)	83.3	<0.001	0.753
>7	13	4.33(2.76–6.80)	86.7	<0.001	0.034

^**a**^: The variables in the subgroup analyses were ART (experienced vs naive), sex (men vs women), age (below 30 vs 30 and above), marital status (married/cohabiting vs not married), educational level (up to primary vs secondary or above), and number of children (none vs one child or more).

^**b, d**^: 1 is not included in confidence intervals when the digits after decimal are kept three.

^**c**^: 2014 is the median.

### Heterogeneity analysis and publication bias

The results of the heterogeneity test and Egger’s test were outlined in Table 2. We performed the random-effects model to do meta-analyses for studies with heterogeneity (*I*^***2***^ ≥50%), and analyzed the rest using a fixed-effects model. According to Egger’s test and the funnel plot, there was evidence of publication bias in many studies included in the meta-analyses of the association of fertility desire with sex and educational level. (Table 2, S1–S6 Figs)

## Discussion

In our study, the prevalence of fertility desire among HIV-infected people was 42.04%, which indicated that their desire to have children cannot be ignored. These meta-analyses demonstrate that for PLHIV, ART use, sex, age, marital status, number of children, and education level are all associated with fertility desire. However, many studies, including a meta-analysis, showed that ART use has no association with fertility desire among PLHIV [9, 24, 76, 77]. In effect, as the most efficient treatment for HIV-infected people, ART could improve their overall well-being, suppress the viral load to a great extent, and help them remain optimistic about fertility [42]. Therefore, it is reasonable for PLHIV on ART to have a higher prevalence of the desire for reproduction than their ART-naive counterparts. In contrast to the previous meta-analysis, this study found that HIV-infected men trend to have more fertility desire than HIV-infected women. Although men and women are both required in the reproductive process, women often suffer the most during pregnancy, which might make them more cautious about fertility [27]. In many patrilineal societies such as in South Africa, HIV-infected men’s greater desire to have children than women might result from the inclination to leave something behind, such as lineage, after they pass away [78–80]. Younger PLHIV, as this study showed, have stronger fertility desire than their older counterparts. For one thing, the desire for children is usually strong in young people of reproductive age, irrespective of HIV infection status. For another, older HIV-infected persons might have already achieved their ideal family sizes and thus would not like to bear other children [14, 52]. Likewise, a majority of studies demonstrated that PLHIV with none or fewer children have a higher prevalence of fertility desire [11, 52, 61]. In some countries, such as Ethiopia, it is believed that family life will not be happy and fulfilled without a child [81]. Additionally, the desire to have biological children and a family of a certain size, as is case in many African nations, are significant reasons for fertility intention among HIV-infected women [82]. As this study indicated, PLHIV who are married or cohabiting have more fertility desire than HIV-infected people who are single, widowed, divorced or separated. Married HIV-infected individuals often have stable relationships or regular sexual partners, and thus might have more reliable support for raising children compared to PLHIV who are not married [50]. Within Ugandan society and in many cultures of Sub-Saharan Africa, childbearing plays a critical role in marriages and families, which may explain the greater fertility desire among PLHIV who are married than among those who are not [66]. Normally, better educated people are expected to have increasingly greater access to information, particularly, AIDS prevention knowledge such as the MTCT of HIV. In addition, well-educated people tend to have better jobs and relatively higher incomes which could make them have better access to medical services [83]. In terms of issues of reproduction, women with higher educational levels tend to make independent decisions, which may be related to higher fertility desire [15]. The meta-analysis likewise showed that HIV-infected people with higher educational levels have more fertility desire than their less educated peers.

Since the advent of ART, it has been possible for PLHIV to give birth to healthy children, due to an enhanced quality of life, longer lifespans and reduced MTCT [76]. However, as a predictor of reproductive practices, fertility desire, if increased, would prompt HIV-infected people to have unprotected intercourse, to give birth and to breastfeed their children, which might heighten the risk of infecting their partners, spouses or children with HIV [8, 84–86]. Many studies have revealed that in Africa horizontal and vertical transmission remain the primary forms of HIV infection, with transmission rates ranging from 20%-25% by HIV-positive people to their HIV-negative partners [87, 88]. Despite being aware of HIV transmission, For many HIV-infected individuals, strong fertility desire could pose barriers to contraception use and thus they would be at risk of unprotected sex [89]. Meanwhile, they would face a complex decision about whether to fulfill their desire to have children.

Consequently, it is important for local health care providers to develop appropriate reproductive health service policies and interventions for men and women living with HIV. For instance, HIV-infected individuals should be provided with counseling and related tests of HIV, such as tests of HIV viral load and CD4 count, and with advice on safe conception. Eligible HIV-infected individuals should initiate ART as soon as possible, and the use of assisted reproductive technology should be recommended for sero-discordant couples. On the whole, fully understanding the fertility desire of PLHIV is essential to providing targeted sexual and reproductive health services.

This study has many limitations. First, there were only six variables included. Previous studies have shown that the following factors are also related to fertility desire among PLHIV: health status, the duration of ART, the influence of partners or spouses, ethnicity, income, culture, stigma, and attitudes of local health care providers; we were unable to extracted these factors either due to a lack of or the unfitness of data for the meta-analyses [18]. As a consequence, it was not easy to draw conclusion on fertility desire by taking six limited variables as determinants. Further, it was found that the duration of ART has potential influence on fertility desire [33]. Therefore it seems more reasonable to consider duration of ART as a factor rather than ART use. Second, there might be a difference in the definition of educational status in various countries; as a result, the pooled effect size of the association between fertility desire and educational level might not be accurate. Moreover, both the lower limit (1.003) of CI in the analysis of ART and the upper limit (0.998) of CI in the analysis of educational level were close to 1, which suggests the need for deeper investigation. Third, the fertility practices of PLHIV are affected by many social factors, such as discrimination; therefore studies on fertility desire might not reflect their genuine intentions. Fourth, the included studies in these meta-analyses were mainly from Sub-Saharan Africa and few were from high-income countries. Hence, positive association of fertility desire with the factors considered in this study is less likely to represent the countries in all of the included studies.

## Conclusion

In summary, the results of this study demonstrate that fertility desire among PLHIV is associated with ART experience, sex, age, marital status, the number of children, and educational level. HIV-infected individuals who are on ART, are male, are younger than 30, are married/cohabiting, are childless and have a secondary education or above have a higher prevalence of fertility desire. Through reproductive health counseling and care, further measures to prevent the horizontal and vertical transmission of HIV should be taken. In addition, their reproductive needs should be met, particularly targeted PLHIV.

## Supporting information

S1 Table PRISMA 2009 checklist. (DOC)

S2 Table Search for PubMed, Cochrane Library, Web of Science and ScienceDirect. (DOCX)

S3 Table Scoring criteria for the quality of studies. (XLSX)

S4 Table Quality assessment of the included studies. (XLSX)

S1 Fig Funnel plot of publication bias of the studies included in the analysis of the association of fertility desire with ART. (PNG)

S2 Fig Funnel plot of publication bias of the studies included in the analysis of the association of fertility desire with sex. (PNG)

S3 Fig Funnel plot of publication bias of the studies included in the analysis of the association of fertility desire with age. (PNG)

S4 Fig Funnel plot of publication bias of the studies included in the analysis of the association of fertility desire with marital status. (PNG)

S5 Fig Funnel plot of publication bias of the studies included in the analysis of the association of fertility desire with educational level. (PNG)

S6 Fig Funnel plot of publication bias of the studies included in the analysis of the association of fertility desire with the number of children. (PNG)
