# Supplementary material for: Prevalence and factors associated with fertility desire among people living with HIV: A systematic review and meta-analysis
Source: PLoS One. 2021 Mar 18;16(3):e0248872. doi: 10.1371/journal.pone.0248872 (PMC7971888; doi:10.1371/journal.pone.0248872)
Supplement: S2 Table — (DOCX) [file pone.0248872.s002.docx]

**Pubmed**

| **Search** | **Add to Builder** | **Query** | **Results** |
| --- | --- | --- | --- |
| #5 |  | Search: #3 AND #4 | 817 |
| #4 |  | Search: ("2000/01/01"[Date - Publication] : "2019/11/24"[Date - Publication]) | 16,928,954 |
| #3 |  | Search: #1 AND #2 | 1188 |
| #2 |  | Search: ((((((((((((Fertility desire*[Title/Abstract]) OR (Fertility intention*[Title/Abstract])) OR (desire to have children[Title/Abstract])) OR (Reproductive intention*[Title/Abstract])) OR (Reproductive decision making[Title/Abstract])) OR (Desire for child[Title/Abstract])) OR (Childbearing desire*[Title/Abstract])) OR (Childbearing intention*[Title/Abstract])) OR (parenthood[Title/Abstract])) OR (fatherhood[Title/Abstract])) OR (motherhood[Title/Abstract])) OR (maternity[Title/Abstract])) OR (paternity[Title/Abstract]) | 41,518 |
| #1 |  | Search: (((HIV[Title/Abstract]) OR (people living with HIV[Title/Abstract])) OR (HIV-positive[Title/Abstract])) OR (HIV-infected[Title/Abstract]) | 315,798 |

**Web of Science**

| **Set** | **Results** | **Web of Science Core Collection**  **Search History**  **Search History -** |
| --- | --- | --- |
| #3 | 1,647 | 1# AND 2# |
| #2 | 58,178 | TS=(Fertility desire OR Fertility intention OR desire to have children OR Reproductive intention OR Reproductive decision making OR Desire for child OR Childbearing desire OR Childbearing intention OR parenthood OR fatherhood OR motherhood OR maternity OR paternity)  *Indexes=SCI-EXPANDED, SSCI, A&HCI, CPCI-S, CPCI-SSH, BKCI-S, BKCI-SSH, ESCI, CCR-EXPANDED, IC Timespan=2000-2019* |
| #1 | 297,136 | TS=(HIV OR people living with HIV OR HIV-positive OR HIV-infected)  *Indexes=SCI-EXPANDED, SSCI, A&HCI, CPCI-S, CPCI-SSH, BKCI-S, BKCI-SSH, ESCI, CCR-EXPANDED, IC Timespan=2000-2019* |

**Cochrane library**

Search Name: 考科兰检索

Date Run: 24/11/2019 14:24:46

Comment:

| **ID** | **Search** | **Hits** |
| --- | --- | --- |
| #1 | (Fertility desire):ti,ab,kw OR (Fertility intention):ti,ab,kw OR (desire to have children):ti,ab,kw OR (Reproductive intention):ti,ab,kw OR (Reproductive decision making):ti,ab,kw | 1468 |
| #2 | (Desire for child):ti,ab,kw OR (Childbearing desire):ti,ab,kw OR (Childbearing intention):ti,ab,kw OR (parenthood):ti,ab,kw OR (motherhood):ti,ab,kw | 1682 |
| #3 | (motherhood):ti,ab,kw OR (maternity):ti,ab,kw OR (paternity):ti,ab,kw | 2135 |
| #4 | #1 OR #2 OR #3 | 4540 |
| #5 | HIV):ti,ab,kw OR (people living with HIV):ti,ab,kw OR (HIV-positive):ti,ab,kw OR (HIV-infected):ti,ab,kw | 25645 |
| #6 | #4 AND #5 | 184 |

**ScienceDirect**

(Fertility desire OR Fertility intention OR desire to have children OR Reproductive intention OR Reproductive decision making OR Desire for child OR Childbearing desire OR Childbearing intention OR parenthood OR fatherhood OR motherhood OR maternity OR paternity) AND (HIV OR people living with HIV OR HIV-positive OR HIV-infected)
