# Supplementary figures and images for: Prevalence and factors associated with fertility desire among people living with HIV: A systematic review and meta-analysis
Source: PLoS One. 2021 Mar 18;16(3):e0248872. doi: 10.1371/journal.pone.0248872 (PMC7971888; doi:10.1371/journal.pone.0248872)

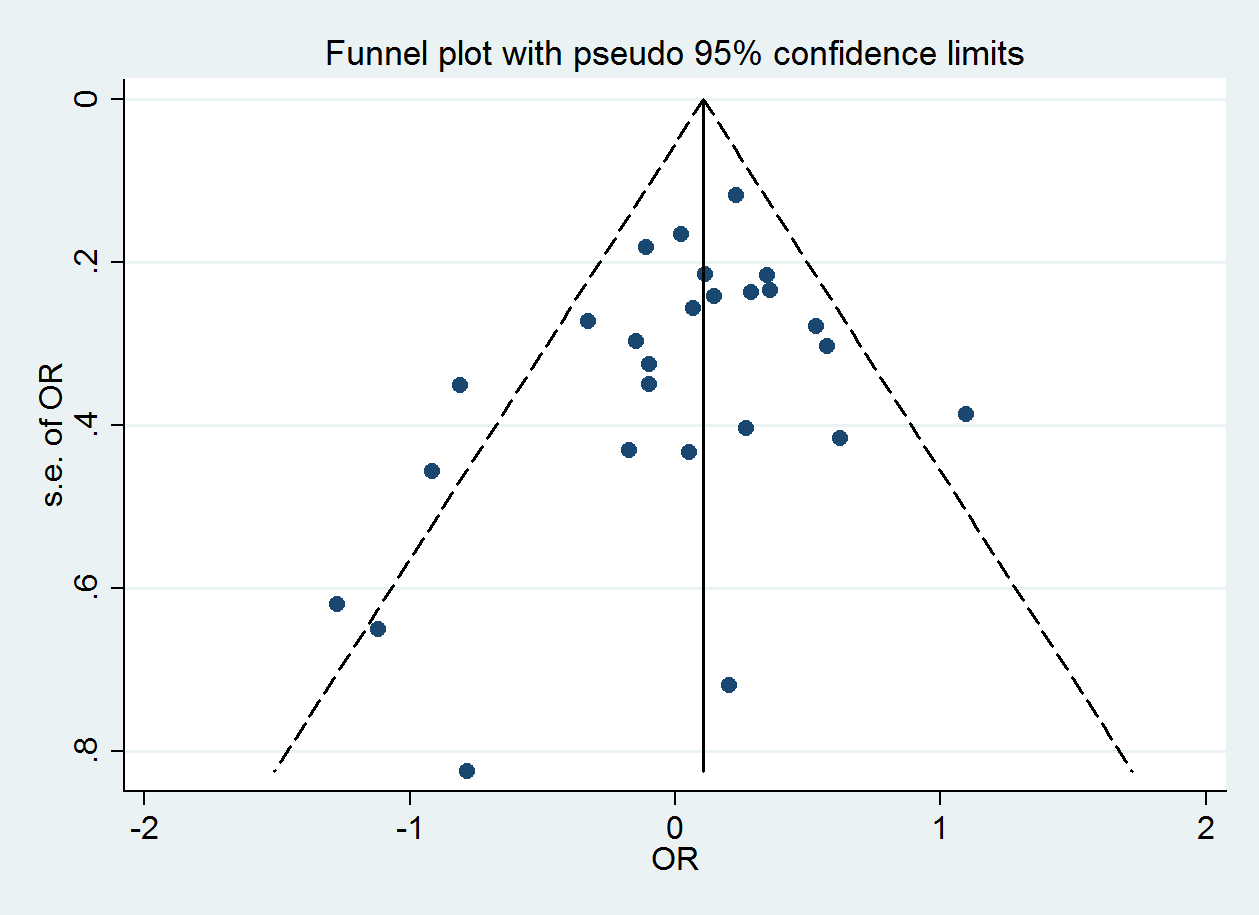

Supplement: S1 Fig — (PNG) [file pone.0248872.s005.png]

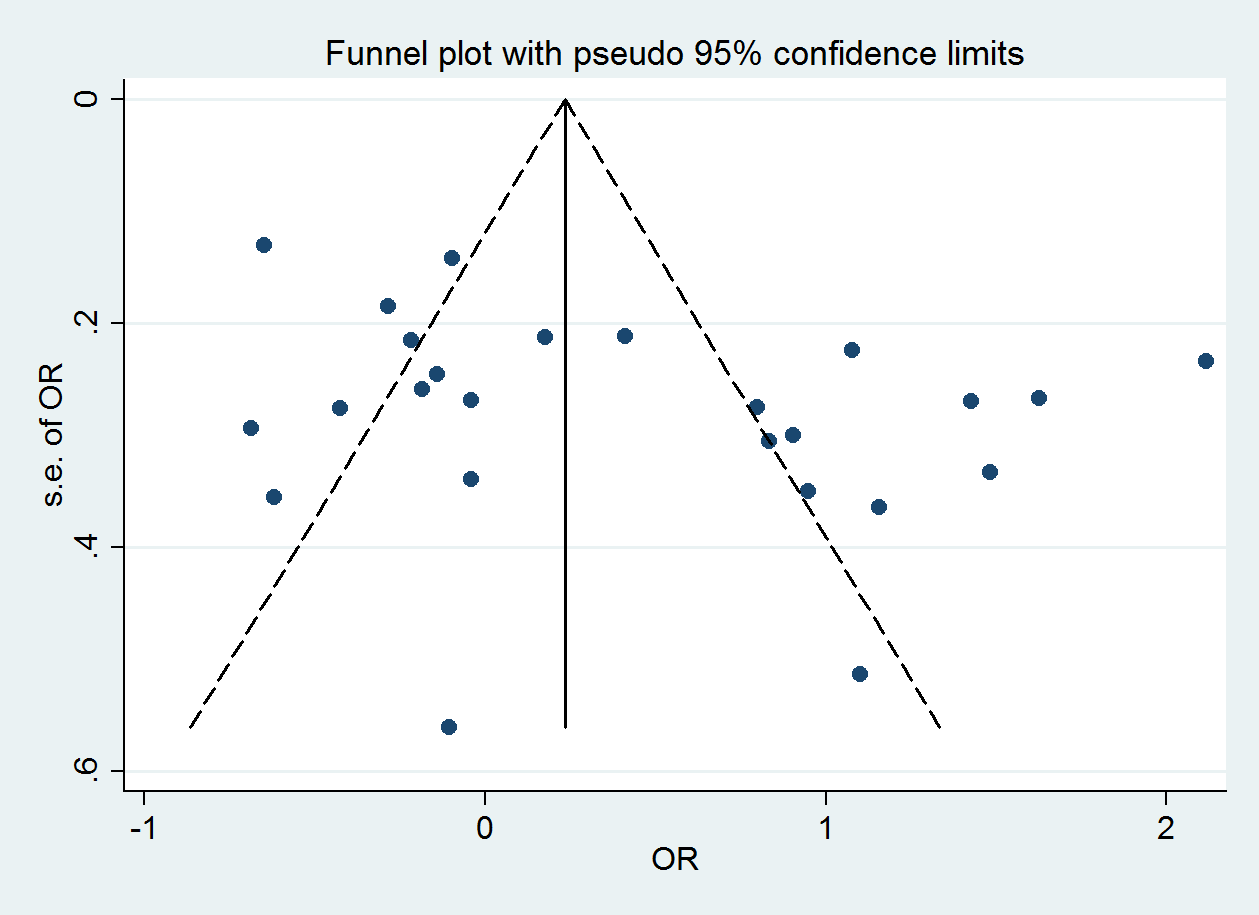

Supplement: S2 Fig — (PNG) [file pone.0248872.s006.png]

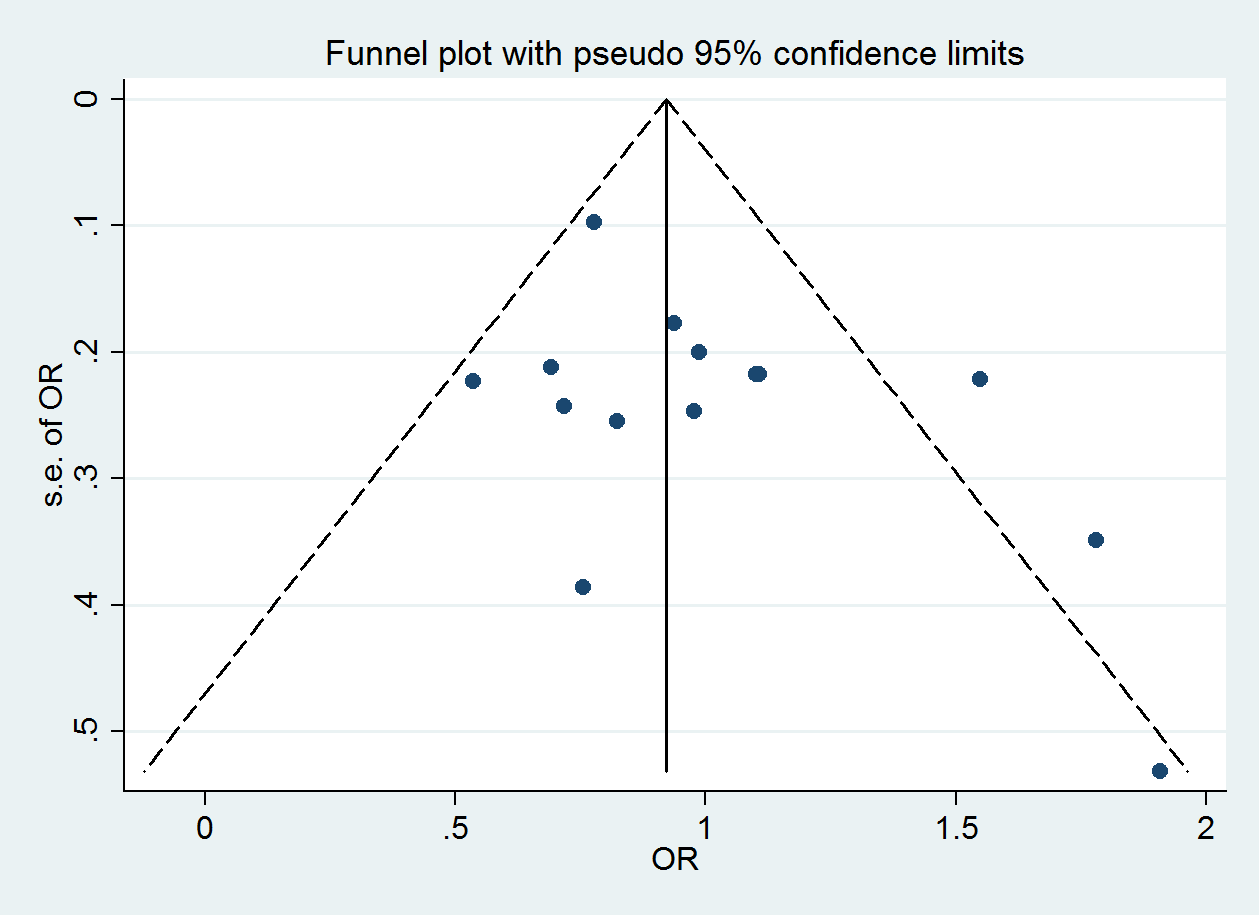

Supplement: S3 Fig — (PNG) [file pone.0248872.s007.png]

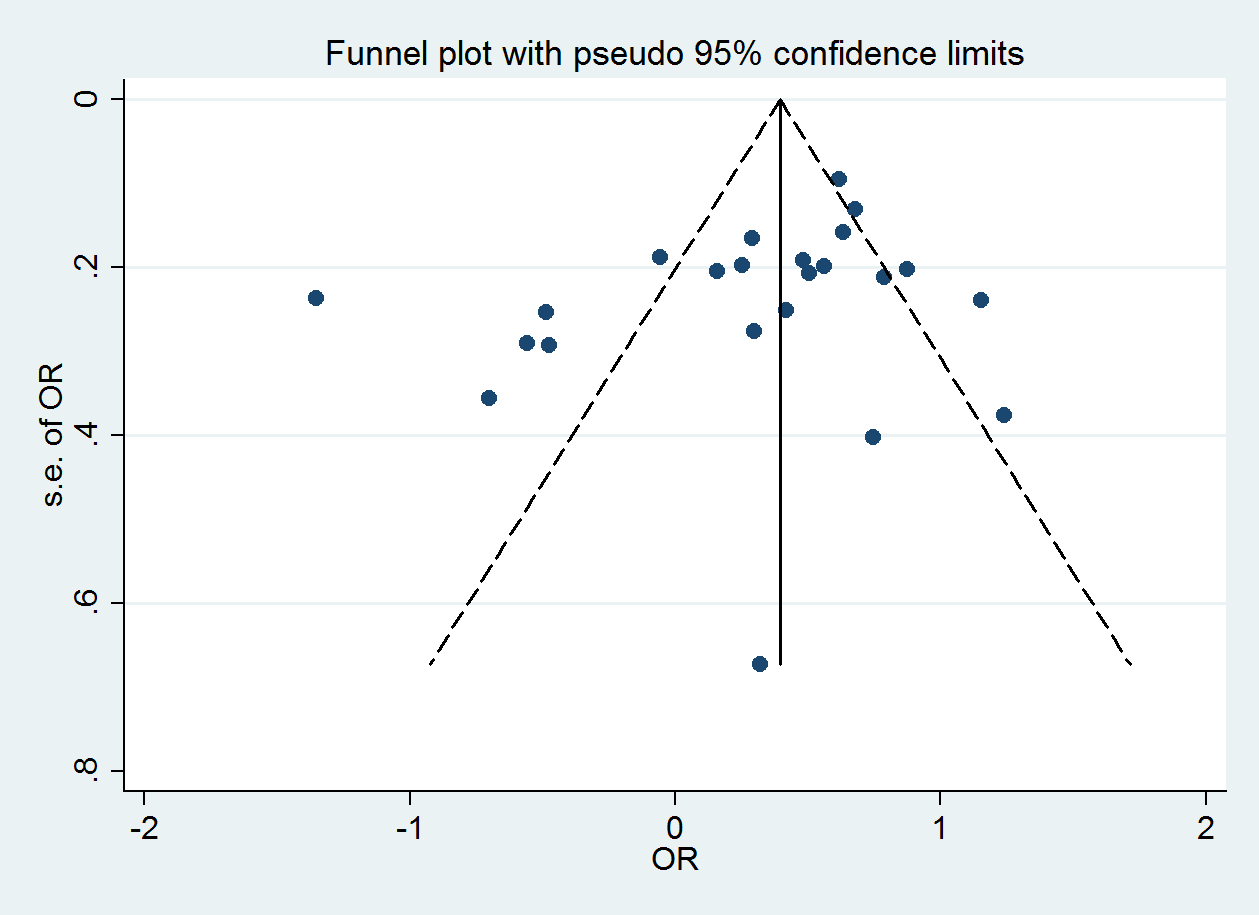

Supplement: S4 Fig — (PNG) [file pone.0248872.s008.png]

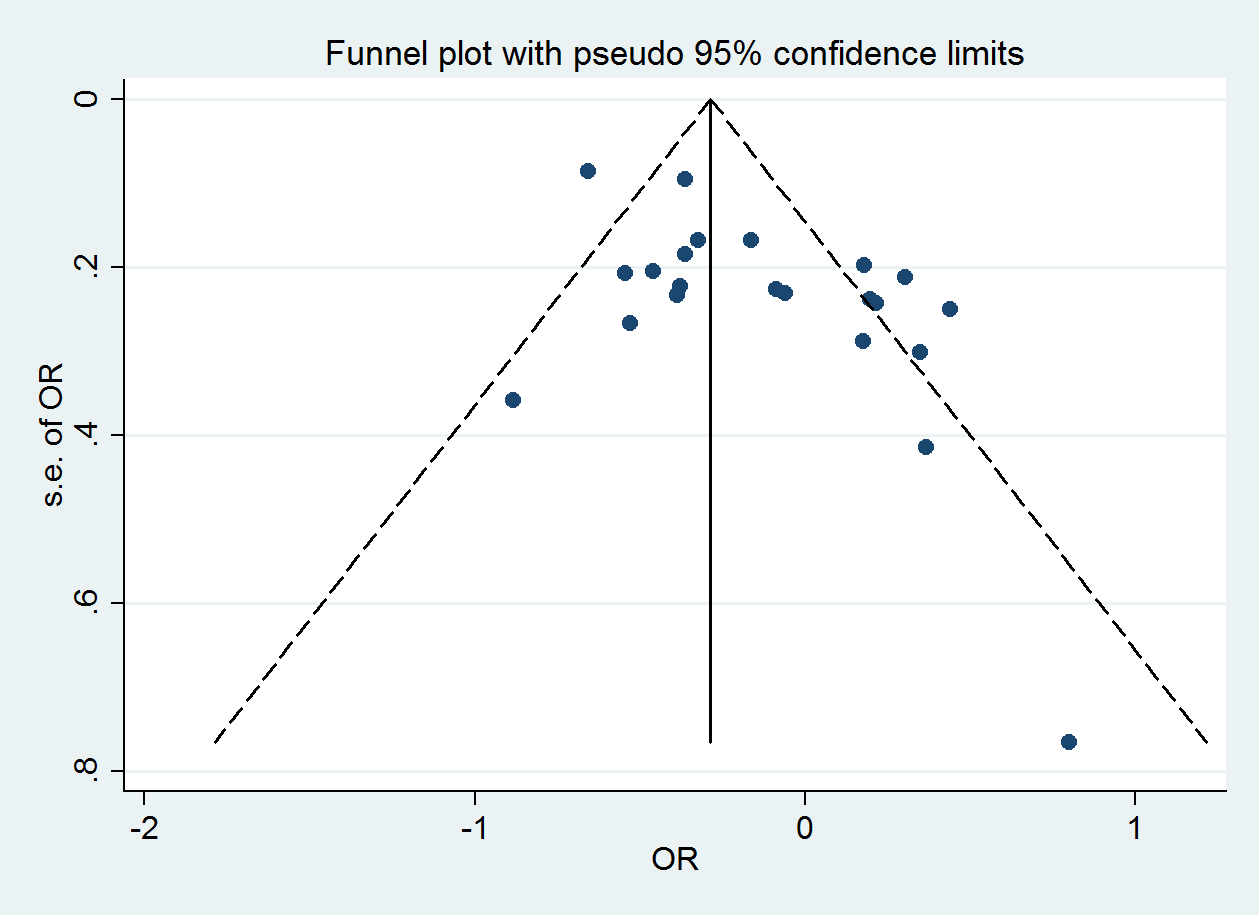

Supplement: S5 Fig — (PNG) [file pone.0248872.s009.png]

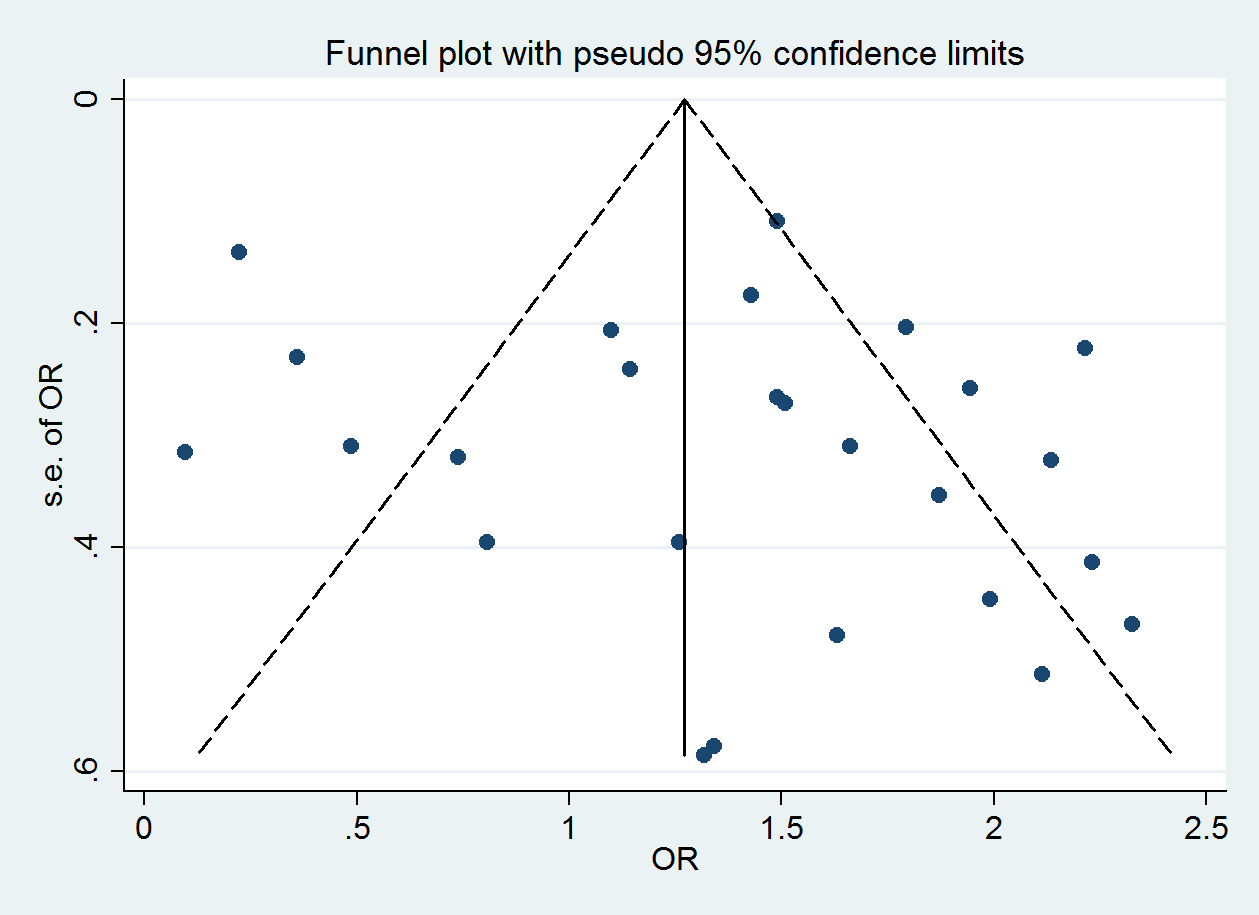

Supplement: S6 Fig — (PNG) [file pone.0248872.s010.png]
